# The Prognostic Value and Potential Mechanism of Tumor-Nutrition-Inflammation Index and Genes in Patients with Advanced Lung Cancer

**DOI:** 10.1155/2023/8893670

**Published:** 2023-05-18

**Authors:** Huan Wang, Yuting Shi, Yueli Shi, Mengqing Cao, Long Zhang, Yuan Wu, Yun Xu, Kai Wang, Xianwu Weng

**Affiliations:** ^1^Department of Respiratory and Critical Care Medicine, The Fourth Affiliated Hospital, Zhejiang University School of Medicine, Yiwu, China; ^2^Department of Cardiothoracic Surgery, The Fourth Affiliated Hospital Zhejiang University School of Medicine, Yiwu, China

## Abstract

**Background:**

Lung cancer (LC) has the highest mortality rate all over the world. It is necessary to search for novel potential biomarkers that are easily accessible and inexpensive in identifying patients with LC at early stage.

**Methods:**

A total of 195 patients with advanced LC who have received first-line chemotherapy were involved in this study. The optimized cut-off values of AGR and SIRI (AGR = albumin/globulin; SIRI = neutrophil *∗* monocyte/lymphocyte) were determined by survival function analysis based on R software. COX regression analysis was performed to obtain the independent factors for establishing the nomogram model. A nomogram model comprising these independent prognostic parameters was built for the TNI (tumor-nutrition-inflammation index) score calculation. The predictive accuracy was demonstrated through ROC curve and calibration curves after index concordance.

**Results:**

The optimized cut-off values of AGR and SIRI were 1.22 and 1.60, respectively. It was revealed that liver metastasis, SCC, AGR, and SIRI were independent prognostic factors in advanced lung cancer by Cox analysis. Afterwards, the nomogram model comprised of these independent prognostic parameters was built for TNI scores calculation. Based on the TNI quartile values, patients were divided into four groups. And it was indicated that higher TNI had worse OS (*P* < 0.05) via Kaplan–Meier analysis and log-rank test. Moreover, the C-index and 1-year AUC area were 0.756 (0.723–0.788) and 75.62, respectively. There was high consistency shown in the calibration curves between predicted and actual survival proportions in the TNI model. In addition, tumor-nutrition-inflammation index and genes play an important role in LC development that might affect some pathways related to tumor development including cell cycle, homologous recombination, and P53 signaling pathway from a molecular level.

**Conclusion:**

TNI might be an analytical tool which was practical and precise for survival prediction of patients with advanced LC. Tumor-nutrition-inflammation index and genes play an important role in LC development. A preprint has previously been published [1].

## 1. Introduction

Lung cancer (LC) which has the highest mortality rate worldwide is the third most common cancer type behind breast and prostate cancers [1, 2]. Most LC patients are diagnosed at an advanced stage with a poor prognosis and short survival time. Therefore, it is vital to explore potential biomarkers that may predict survival time and identify patients who may benefit from early treatment. Immune checkpoints are widely accepted biomarkers in immunotherapy, including programmed cell death protein 1/programmed cell death 1-ligand 1, and cytotoxic T-lymphocyte-associated protein 4. Other biomarkers such as EGFR, RAS, and TP53 are widely used in target therapy for LC. However, invasive procedures were required to detect these biomarkers including obtaining pathological tissues that are costly. Therefore, it is optimal to identify potential biomarkers that are easily accessible and inexpensive.

Laboratory blood tests, including various indicators such as absolute white cell counts, albumin, globulin, neutrophils, monocytes, and lymphocytes, are widely used in clinical practice. Previous studies have suggested that these blood indicators could be used as predictive and prognostic biomarkers for various tumors, including LC [3–5]. And albumin and globulin are the most common clinical nutritional indicators. Moreover, inflammatory indicators, including neutrophils, monocytes, and lymphocytes, usually can be used to reflect the inflammatory state. Besides, it has been reported that low AGR or high SIRI is associated with poor survival outcomes [6–8]. Nutritional and inflammatory indicators usually are related to the prognosis of patients with cancer [2, 9, 10]. Nevertheless, there are some limitations on these studies, including focusing on a single marker, patients at a certain stage, and a particular cytological classification. Few studies have investigated the association between combined factors and the prognosis of advanced LC. This study aimed to explore the prognostic significance and potential mechanisms of integrated nutritional and inflammatory values and genes in patients with advanced LC.

## 2. Patients and Methods

### 2.1. Patients

A retrospective analysis was conducted those enrolled patients with a definite diagnosis of stage IV LC treated in the respiratory medicine department of the Fourth Affiliated Hospital of Zhejiang University School of Medicine in the past 5 years from February 2015 to December 2019. The inclusion and exclusion criteria were as follows:

Inclusion criteria were at least 18 years of age with a definite diagnosis of stage IV LC by CT or MRI imaging examination and pathological examination, initial first-line chemotherapy was treated in the Fourth Affiliated Hospital; all patients were ECOG PS 0-1; complete clinical and follow-up information; and sufficient pretreatment routine blood laboratory test data.

Exclusion criteria were patients without a diagnosis of LC; patients with repeat names and hospital admission number; without complete clinical information; without sufficient follow-up information; no available routine blood laboratory data; and no history of acute infection.

The study was approved by the Research Ethics Committee of the Fourth Affiliated Hospital of Zhejiang University School of Medicine (reference number: K2021063).

### 2.2. Data Collection

Clinical information was collected from the electronic medical record system. CT or MRI imaging and pathology examinations were performed by at least two professional physicians. Laboratory test data were selected within two weeks prior to the first-line chemotherapy by detecting the patient's peripheral blood.

### 2.3. Follow-Up

All patients were followed up every three months. The estimated endpoints were the overall survival (OS). OS was defined as the interval from the start of first-line chemotherapy up to the time of the patient's last follow-up or the time of death. All patients were followed up to January, 2021.

### 2.4. The Evaluation of AGR and SIRI

The albumin, globulin, neutrophil, monocyte, and lymphocyte values were collected to calculate the AGR and SIRI (AGR = albumin/globulin; SIRI = neutrophil *∗* monocyte/lymphocyte). The optimized cut-off values were dichotomized through survival function using R software 3.6.2. According to the optimized cut-off value, all included patients were classified into elevated and low groups. The cut-off values were 1.22 and 1.60, respectively.

### 2.5. The Analysis of Biological Functions and Pathways

The dataset was downloaded from TCGA-LUAD (https://portal.gdc.cancer.gov/) which included 522 LUAD patients with complete clinical information, survival information, gene expression, etc. A total of 389 CRP-ALB related-genes was obtained from the website https://www.gsea-msigdb.org/gsea, the neutrophil-related gene set was received from the dataset of HP_ABNORMALITY_OF_NEUTROPHILS, and the albumin-related gene set was downloaded from the dataset of HP_HYPOALBUMINEMIA. Differential genes between tumor and nontumor tissues were identified by limma package and the criteria of *P* < 0.05. The dependent risk prognostic genes were screened by the univariate and multivariate Cox regression and LASSO regression using the survival and glmnet package of R studio software. KEGG functional enrichment analysis was performed to explore the potential mechanisms.

### 2.6. Statistical Analysis

All statistical analyses were performed using R software (R 3.6.2 version), SPSS software (IBM SPSS statistical 20.0 version), and GraphPad software (GraphPad Prism 6 version). All count data were extracted as continuous variable values or percentage values. Chi-square test, Fisher's exact test, and Bonferroni correction were used to compare categorical variables. Kaplan–Meier (KM) survival curves and log-rank tests were used to explore the distribution of the OS of categorical variables. Univariate and multivariate Cox regression analyses were performed to analyze the significant independent prognostic factors. The statistical significance threshold was set to *P* value of less than 0.05.

## 3. Results

### 3.1. Patients Selection

**Figure 1:**
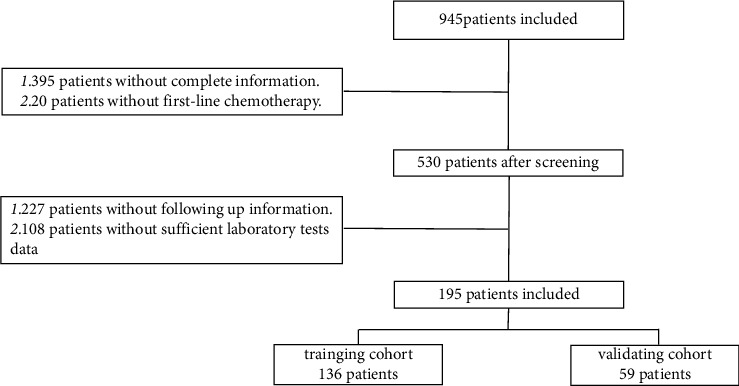
Flowchart of study selection process.

This retrospective analysis study included 945 patients with LC initially, who were hospitalized in the respiratory medicine department of the Fourth Affiliated Hospital of Zhejiang University School of medicine in the past 5 years from February 2015 to December 2019. Based on the inclusion and exclusion criteria, 395 patients were without complete information, 20 patients were without first-line chemotherapy, 277 patients were without follow-up information, and 108 patients were without sufficient laboratory test data. Finally, 195 patients were included in this retrospective study. In addition, R software was used to randomly group the patients to a 7 : 3 ratio. Finally, the nomogram prediction model was established based on the training group including 136 patients. And other 59 patients were assigned to the validation cohort. The total patients were assigned to the testing cohort to assess the model (Figure 1).

### 3.2. Association between AGR, SIRI, and the OS

The characteristic variables of the training cohort are summarized in Table 1. The median value of age was regarded as the cut-off value. The cut-off values of CRP, CEA, and CA199 are defined by the maximum of the normal range setting by the Fourth Affiliated Hospital of Zhejiang University School of Medicine. The training cohort consisted of 96 (70.6%) men and 40 (29.4%) women. In addition, Table 1 shows that low AGR is significantly associated with other prognostic outcomes, including no history of LC operation (*P*=0.005), body mass index (BMI) of ≥18.5 (*P*=0.008), carcinoembryonic antigen (CEA) of ≥5 (*P*=0.018), and an increased C-reactive protein (CRP) level (*P* < 0.001). It was significantly different when comparing high SIRI with gender (*P*=0.002), pathology (*P*=0.008), and CRP (*P* < 0.001).

**Figure 2:**
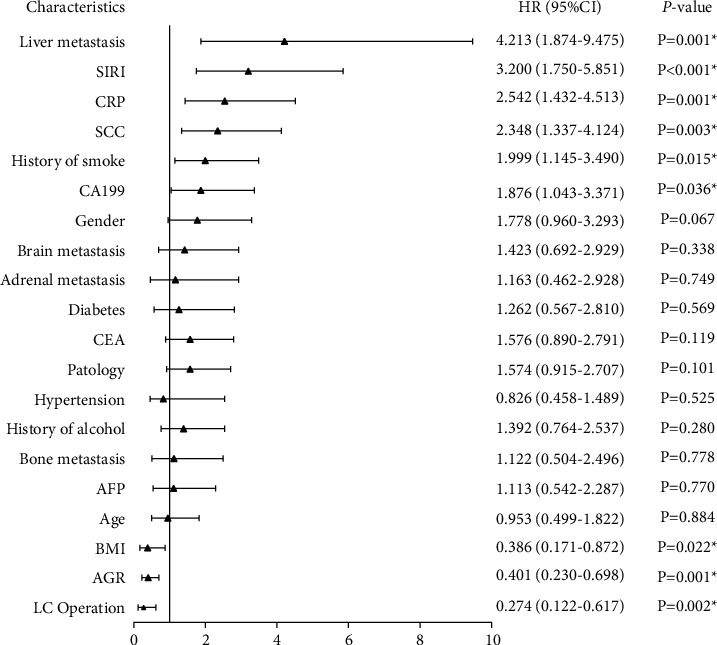
Forest of COX univariate regression analysis in training cohort.

**Figure 3:**
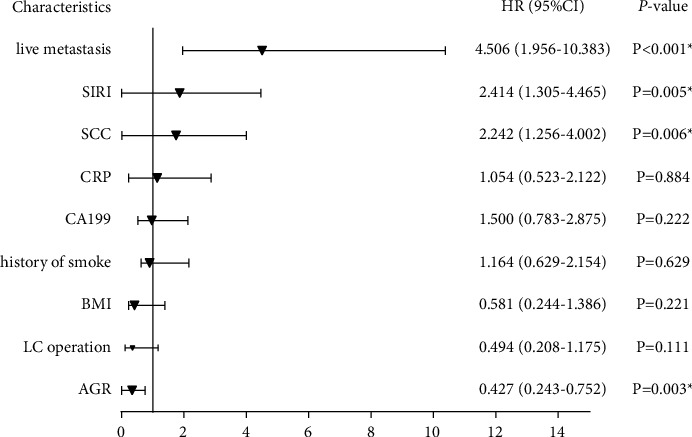
Forest of COX multivariate regression analysis in training cohort.

Subsequently, Cox univariate and multivariate regression analyses included variables that were significant in Table 1 or meaningful related clinical work. It was indicated that a history of LC operation, liver metastasis, history of smoking, BMI, CA199, squamous cell carcinoma antigen (SCC), CRP, AGR, and SIRI were significantly associated with OS (*P* < 0.05; Figure 2). And it was revealed that liver metastasis, SCC, AGR, and SIRI were independent prognostic factors in advanced LC through Cox multivariate proportional hazard analysis (*P* < 0.05; Figure 3).

**Figure 4:**
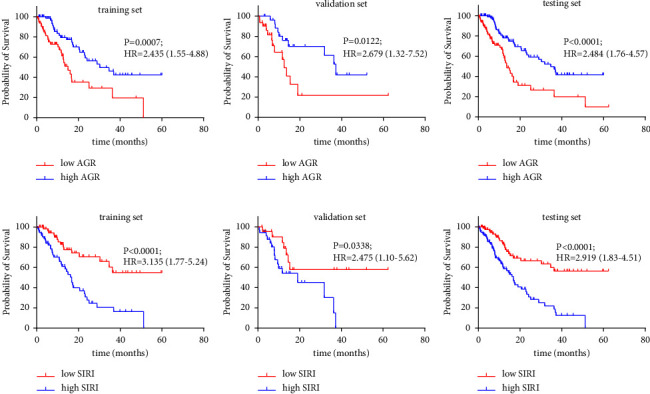
Kaplan–Meier curves for OS according to the optimized cut-off value about albumin to globulin (AGR) and neutrophil *∗* monocyte/lymphocyte (SIRI). (a) AGR in the training cohort; (b) AGR in the validation cohort; (c) AGR in the testing cohort; (d) SIRI in the training cohort; (e) SIRI in the validation cohort; (f) SIRI in the total cohort.

To explore the prognostic value of AGR and SIRI in patients with advanced LC, KM analysis and log-rank test demonstrated that the relationship between low AGR and poorer OS was statistically significant in the training set (hazard ratio [HR] = 2.435 [1.55–4.88], *P*=0.007; Figure 4(a)). The lower AGR group had shorter 5-year OS rate (0% vs. 42.3%) and median OS time (15.0 months vs. 30.3 months) in comparison with the elevated AGR level group. When patients with advanced LC were in hyperinflammatory states, it revealed that high SIRI level had lower 5-year OS rate (0% vs. 54.9%) and median OS time (16.7 months vs. NA; HR = 3.135 (1.77–5.24); *P* < 0.001; Figure 4(d)). Similar results were confirmed in the validation and testing sets (*P* < 0.05; Figures 4(b) and 4(c)-4(e) and 4(f)).

### 3.3. The Analysis of the Prognostic Value of TNI

**Figure 5:**
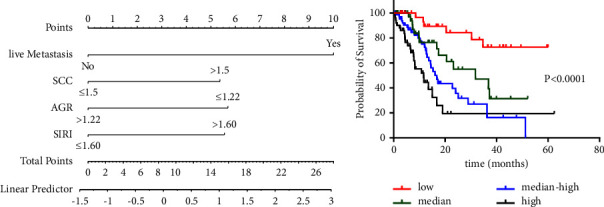
Nomogram model and Kaplan–Meier curve about TNI in training cohort: (a) nomogram to calculate risk score and predict survival probability and (b) predicted patient survival probability curve corresponding to different risk ranging from low risk to high risk according to the quarter of the TNI risk scores.

The potential value of the clinical factors in the training set was further explored. As known, SCC and liver metastasis are important biomarkers for LC screening [11, 12]. However, not all patients with high SCC or liver metastasis have a poor survival time. SCC or liver metastasis alone is insufficient as a prognostic biomarker for patients with advanced LC. Therefore, more prognostic biomarkers for LC need to be explored. To predict survival precisely and quantitatively, a nomogram model based on relevant parameters was established. The total points were calculated by determining the score of the parameters by establishing the nomogram as shown in Figure 5(a). Liver metastasis had the largest interval while the SCC risk score indicated the minimum range in this model. The total point was defined as the TNI, which was calculated for each patient based on the model. We could get a formula:TNI = 10 *∗* liver metastasis^yes^ + 5.37 *∗* SSC^high^ + 5.69 *∗*AGR^low^ + 5.55 *∗* SIRI^high^. TNI scores were calculated using the R software for each patient with advanced LC. Then, all patients included were divided into four groups based on their TNI quartile values. KM analysis and log-rank test indicated that the high-risk TNI group significantly predicted poorer OS compared to the other groups, as shown in Figure 5(b) (*P* < 0.05).

To verify whether the nomogram model is applicable to both the training and validation sets, the TNI score for each patient was disposed in the same manner as the testing set. The survival curves were still statistically significant, as plotted in Supplementary Figures 1A and 1B (*P* < 0.05). In order to further validate the diagnostic ability of the nomogram model, the concordance index (C-index) and time-dependent receiver characteristic operator (ROC) curves were drafted by R studio according to the SCC combined liver metastasis model, AGR combined SRI model, and TNI model, respectively. The results showed that the C-index was 0.658 (0.621–0.694), 0.703 (0.666–0.739), and 0.756 (0.723–0.788), respectively. The 1-year AUC areas were 68.93, 67.34, and 75.62, respectively (Supplementary Table 1; Supplementary Figure 1C). This demonstrated that TNI had a higher diagnostic ability than the other two models. It showed elevated consistency for comparing predicted and actual survival proportions for the TNI model in the training, validation, and testing sets, which were revealed by calibration curves at 1 year, 2 years, and 3 years (Supplementary Figures 2A–2I).

**Figure 6:**
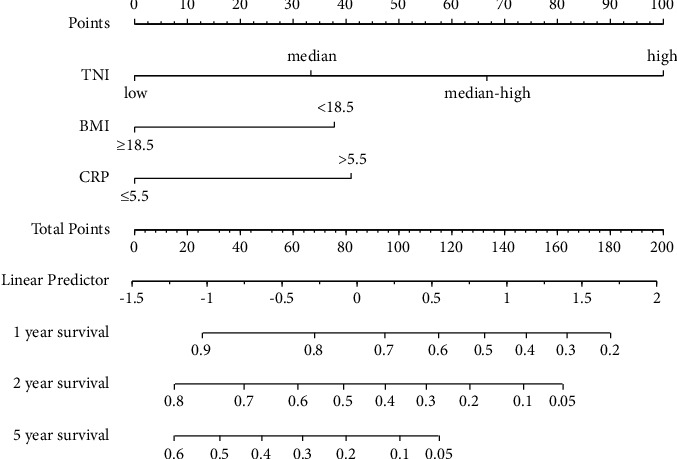
The nomogram prognostic model was built based on the TNI in patients with advanced lung cancer in total population.

Based on the TNI scores, the patients' clinical characteristics in the total population are shown in Supplementary Table 2. We then performed Cox univariate and multivariate regression analyses, as shown in Supplementary Table 3. BMI, CRP, and TNI were independent prognostic factors in patients with advanced LC (*P* < 0.05). A nomogram prognostic model was established to predict survival time rates according to these three independent risk factors in the total population (Figure 6). Using the prognostic model, we can intuitively observe the survival rate of patients with advanced LC.

The regimen of LC has entered an era of precision treatment, so subgroup analysis was conducted in the LC subtypes, and patients were divided into EGFR-mutation and non-EGFR-mutation groups for exploring the potential significance of TNI. The optimized cut-off value was obtained using the R package as shown in Supplementary Figures 3A and 3B. Both subgroups showed longer survival time in patients with low TNI. Additionally, when patients were separated into chemotherapy and targeted and immunotherapy groups according to the First-line chemotherapy regimen, the results demonstrated that patients with high TNI had worse OS (Supplementary Figures 3C and 3D). So, we could conclude that TNI may be a potential biomarker for patients screening and treatment regimens options influencing.

### 3.4. The Potential Mechanism Exploration of Tumor-Inflammation-Nutrition-Genes

**Figure 7:**
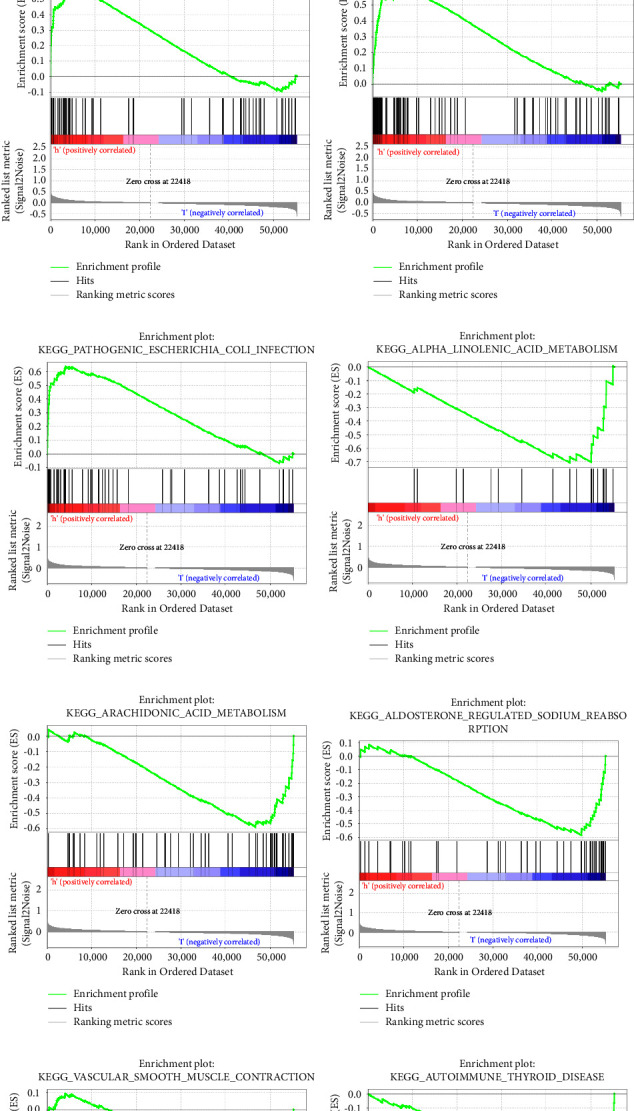
Genomic enrichment analysis of biological pathways. (a) Cell cycle; (b) homologous recombination; (c) P53 signaling pathway; (d) pyrimidine metabolism; (e) pathogenic *Escherichia coli* infection; (f) alpha linolenic acid metabolism; (g) arachidonic metabolism; (h) aldosterone-regulated sodium reabsorption; (i) vascular smooth muscle contraction; (j) autoimmune thyroid disease.

The results above of this study showed that inflammation and nutritional levels play a crucial role in tumor development, so we explored the potential mechanisms of neutrophil- and albumin-related genes affecting lung cancer. There were 11 independent prognostic tumor-neutrophil-albumin-associated genes (AK2, BTK, DMD, DSG2, EIF2AK3, PIK3CG, PRKCD, RFXAP, ANLN, MYO1E, OSGEP) by differential genes screening, univariate and multivariate COX regression analyses, and LASSON regression (Table 2). Then, we performed pathway enrichment analysis using the genes above, and the results indicated that these differential tumor-neutrophil-albumin-associated genes may be involved in the following pathways: cell-cycle, homologous recombination, P53 signaling pathway, pyrimidine metabolism, pathogenic *Escherichia coli* infection, alpha linolenic acid metabolism, arachidonic metabolism, aldosterone-regulated sodium reabsorption, vascular smooth muscle contraction, autoimmune thyroid disease (Figures 7(a)–7(j)). The p53 protein is a nuclear transcription factor that regulates the expression of a wide variety of genes involved in apoptosis, growth arrest, or senescence in response to genotoxic or cellular stress. Abnormal cell cycle and homologous recombination could cause over proliferation of cells and an accumulation of abnormal cell numbers. So, the genes above may influence some tumor signal pathways during tumor progression to a great extent.

## 4. Discussion

In this study, we found that liver metastasis, SCC, AGR, and SIRI were independent significant prognostic factors in patients with advanced LC that is consistent with previous studies [8, 13]. Liver metastasis and SCC are widely accepted biomarkers in clinical studies. AGR has been reported to be related to long-term survival in various tumors, and it has been suggested that AGR is an independent prognostic factor [7, 14]. Previous meta-analysis including 12 studies on AGR and gastric cancer outcomes demonstrated that a higher AGR was associated with longer survival time [15]. SIRI has also been demonstrated as an effective prognostic biomarker for solid tumors, including stage III nonsmall cell LC [8, 16–19]. However, it is still controversial to utilize these indicators as independent prognostic biomarkers. On the one hand, the correlation among the variables in the prediction model was demonstrated via the nomogram of TNI, which is based on multifactor regression analysis to integrate multiple predictors and then use scaled line segments, drawn on the same plane according to a certain scale. Afterwards, complex regression equations were transformed into visual graphs that make the results of the prediction model more readable, convenient for patient assessment, and intuitive, and it is also easy to understand in medical research and clinical practice. On the other hand, similar nomograms have been used in the past to evaluate the prognostic prediction of tumors [20, 21]. However, there is no such model for TNI. Therefore, TNI may be a potential biomarker for effectively and precisely predicting the survival rate of patients with advanced LC. It makes a significant contribution to the literature because it is the first to combine nutritional, inflammatory, and clinical indicators to establish an integrated biomarker and nomogram model for predicting survival outcomes. This provides a practical analytical tool for more accurate prediction of survival outcomes in patients with advanced LC.

Albumin, synthesized in the liver, is considered the most essential protein in human plasma. Several bodily activities were maintained by albumin, including nutrition and osmotic pressure, transporting and binding hormones, pharmaceuticals, fatty acids, and cations [13]. Close relationship was indicated between serum albumin level and nutritional status. Some previous studies have revealed that albumin participates in the inflammatory response process [22, 23]. And it was shown that upregulation of albumin promotes tumor proliferation and metastasis via activating expression of tumor necrosis factor-*α*, interleukin-1, and interleukin-6 [24]. Moreover, albumin nanovectors play a crucial role in increasing the availability of drugs and drug delivery, such as albumin paclitaxel [25]. Low albumin levels are associated with poor liver function, and patients with advanced cancer were reported to display a high incidence of malnutrition due to cancer cachexia and cancer-associated bleeding [26]. A large number of immune-related products were contained in globulin, which can trigger antigen binding and recognition, complement activation, and Fc receptor binding by stimulating the lymphatic system [27]. Although it has been confirmed that globulin plays an important role in the immune microenvironment [28, 29], it was still confused whether it could affect tumor immunotherapy or not. Studies have shown that neutrophils can promote tumor metastasis through arachidonate 5-lipoxygenase-dependent leukotriene synthesis [30]. In addition, it can inhibit the activation of CD + T cells and increase the secretion of cathepsin G, neutrophil elastase, and other factors that promote tumor metastasis [31, 32]. Monocytes can differentiate into macrophages or dendritic cells, which are involved in the immune response. Studies have confirmed that the CCL2-CC chemokine receptor 2 signaling pathway can be blocked by reducing the activation and proliferation of monocytes to promote tumor cell metastasis inhibition [33]. Lymphocytes are a type of cell line with immune recognition function, which can be divided into T lymphocytes, B lymphocytes, and natural killer (NK) cells. T lymphocytes, such as CD4+ cells, usually play a crucial role in the tumor immune response including releasing immunoregulatory factors, inhibiting tumor growth and metastasis, and other processes [34]. B lymphocytes and NK cells play a crucial role in tumor immune response and inhibition of tumor proliferation and metastasis through the secretion of tumor-specific antibodies [35, 36]. A valuable prognostic indicator would be produced when the above indicators are combined. In addition, it may affect some pathways related to tumor development including cell cycle, homologous recombination, and P53 signaling pathway from a molecular level. However, whether these genes actually affect lung cancer invasion and metastasis at the molecular level? whether they will comprehensively affect antitumor efficacy? This mechanism needs to be further explored.

This study has several limitations. First, as mentioned, although this study had an external verification of the validation cohort and the testing cohort based on the results of the training set, it is still a single-center retrospective study, and a more multicenter retrospective studies with more patients and high-quality prospective studies are needed in the future. Second, this study only included patients with advanced LC and patients who underwent surgery or had sufficient concurrent infection are not included here. And salvage treatments may have altered the results in favor of one group unintentionally. Third, this study only focused on the evaluation of the nutritional and inflammatory status of patients with advanced LC before first-line chemotherapy. It is still unknown whether the TNI index can be used as an indicator for the dynamic monitoring indicator in the treatment stage since the influence of subsequent chemotherapy or radiotherapy and other antitumor therapies on the overall nutritional inflammation level of patients has not been thoroughly explored. Fourth, the follow-up time was short because patients included in this study started in February 2015. The follow-up time should be extended in future studies to make the results more reliable. Finally, the cut-off value adopted in this study was optimized by calculation using R software. It was still uncertain whether this value can better classify patients, or whether it can be applied in a larger population for business reasons or not.

## 5. Conclusion

In conclusion, to our knowledge, this study firstly combined nutritional, inflammatory, and clinical indicators to establish an integrated biomarker TNI for predicting survival outcomes. It showed that there are some potential molecular mechanisms between neutrophil-albumin-related genes and LC development. A practical analytical tool is provided for more accurate prediction of survival outcomes in patients with advanced LC. This analysis may provide a strong support for the selection of clinical treatment strategies.

## Figures and Tables

**Table 1:** Clinical characteristics of the patients with different AGR and SIRI according to the optimized cut-off value in training dataset.

	No. of patients (*N* = 136)	AGR < 1.22 (*N* = 64)	AGR ≥ 1.22 (*N* = 72)	*P* value	SIRI < 1.60 (*N* = 61)	SIRI ≥ 1.60 (*N* = 75)	*P* value
Gender				0.492			0.002
Female	40 (29.4%)	17 (26.6%)	23 (31.9%)		26 (42.6%)	14 (18.7%)	
Male	96 (70.6%)	47 (73.4%)	49 (68.1%)		35 (57.4%)	61 (81.3%)	
Age				0.089			0.875
≤60	37 (27.2%)	13 (20.3%)	24 (33.3%)		17 (27.9%)	20 (26.7%)	
>60	99 (72.8%)	51 (79.7%)	48 (66.7%)		44 (72.1%)	55 (73.3%)	
History of LC operation				0.005			0.135
No	102 (75.0%)	55 (85.9%)	47 (65.3%)		42 (68.9%)	60 (80.0%)	
Yes	34 (25.0%)	9 (14.1%)	25 (34.7%)		19 (31.1%)	15 (20.0%)	
Differentiation				0.172			0.078
Poor	36 (26.5%)	19 (29.7%)	17 (23.6%)		12 (19.7%)	24 (32.0%)	
Moderately well	13 (9.6%)	3 (4.7%)	10 (13.9%)		9 (14.8%)	4 (5.3%)	
Unknown	87 (64.0%)	42 (65.6%)	45 (62.5%)		40 (65.6%)	47 (62.7%)	
Pathology				0.7			0.008
Adenocarcinoma	72 (52.9%)	35 (54.7%)	37 (51.4%)		40 (65.6%)	32 (42.7%)	
Nonadenocarcinoma	64 (47.1%)	29 (45.3%)	35 (48.6%)		21 (34.4%)	43 (57.3%)	
Mutation				0.822			0.204
Positive	56 (41.2%)	28 (43.8%)	28 (38.9%)		30 (49.2%)	26 (34.7%)	
Negative	41 (30.1%)	19 (29.7%)	22 (30.6%)		17 (27.9%)	24 (32.0%)	
Unknown	39 (28.7%)	17 (26.6%)	22 (30.6%)		14 (23.0%)	25 (33.3%)	
Bone metastasis				0.763			0.179
No	114 (83.8%)	53 (82.8%)	61 (84.7%)		54 (88.5%)	60 (80.0%)	
Yes	22 (16.2%)	11 (17.2%)	11 (15.3%)		7 (11.5%)	15 (20.0%)	
Brain metastasis				0.869			0.596
No	114 (83.8%)	54 (84.4%)	60 (83.3%)		50 (82.0%)	64 (85.3%)	
Yes	22 (16.2%)	10 (15.6%)	12 (16.7%)		11 (18.0%)	11 (14.7%)	
Adrenal metastasis				0.413			0.707
No	124 (91.2%)	57 (89.1%)	67 (93.1%)		55 (90.2%)	69 (92.0%)	
Yes	12 (8.8%)	7 (10.9%)	5 (6.9%)		6 (19.8%)	6 (8.0%)	
Liver metastasis				0.514			0.097
No	123 (90.4%)	59 (92.2%)	64 (88.9%)		58 (95.1%)	65 (86.7%)	
Yes	13 (9.6%)	5 (7.8%)	8 (11.1%)		3 (4.9%)	10 (13.3%)	
History of smoke				0.479			0.112
No	70 (51.5%)	35 (54.7%)	35 (48.6%)		36 (59.0%)	34 (45.3%)	
Yes	66 (48.5%)	29 (45.3%)	37 (51.4%)		25 (41.0%)	41 (54.7%)	
History of alcohol				0.427			0.619
No	102 (75.0%)	50 (78.1%)	52 (72.2%)		47 (77.0%)	55 (73.3%)	
Yes	34 (25.0%)	14 (21.9%)	20 (27.8%)		14 (23.0%)	20 (26.7%)	
Hypertension				0.444			0.264
No	89 (65.4%)	44 (68.8%)	45 (62.5%)		43 (70.5%)	46 (61.3%)	
Yes	47 (34.6%)	20 (31.2%)	27 (37.5%)		18 (29.5%)	29 (38.7%)	
Diabetes				0.606			0.881
No	121 (89.0%)	56 (87.5%)	65 (90.3%)		54 (88.5%)	67 (89.3%)	
Yes	15 (11.0%)	8 (12.5%)	7 (9.7%)		7 (11.5%)	8 (10.7%)	
BMI				0.008			0.407
<18.5	12 (8.8%)	10 (15.6%)	2 (2.8%)		4 (6.6%)	8 (10.7%)	
≥18.5	124 (91.2%)	54 (84.4%)	70 (97.2%)		57 (93.4%)	67 (89.3%)	
CEA				0.018			0.88
≤5	57 (41.9%)	20 (31.2%)	37 (51.4%)		26 (42.6%)	31 (41.3%)	
>5	79 (58.1%)	44 (68.8%)	35 (48.6%)		35 (57.4%)	44 (58.7%)	
CA199				0.078			0.602
≤43	95 (69.9%)	40 (62.5%)	55 (76.4%)		44 (72.1%)	51 (68.0%)	
>43	41 (30.1%)	24 (37.5%)	17 (23.6%)		17 (27.9%)	24 (32.0%)	
SCC				0.113			0.361
≤1.5	88 (64.7%)	37 (57.8%)	51 (70.8%)		42 (68.9%)	46 (61.3%)	
>1.5	48 (35.3%)	27 (42.2%)	21 (29.2%)		19 (31.0%)	29 (38.7%)	
AFP				0.734			0.589
≤7	111 (81.6%)	53 (82.8%)	58 (80.6%)		51 (83.6%)	60 (80.0%)	
>7	25 (18.4%)	11 (17.2%)	14 (19.4%)		10 (16.4%)	15 (20.0%)	
CRP				<0.001			<0.001
≤5.5	63 (46.3%)	14 (21.9%)	49 (68.1%)		40 (65.6%)	23 (30.7%)	
>5.5	73 (53.7%)	50 (78.1%)	23 (31.9%)		21 (34.4%)	52 (69.3%)	

**Table 2:** Independent prognostic genes and corresponding coefficients.

Gene	Coefficients
AK2	−0.018723084599359
BTK	−0.054056140795858
DMD	−0.044402599920014
DSG2	0.028396420400791
EIF2AK3	−0.170817434379354
PIK3CG	−0.043947428587623
PRKCD	−0.155436840113741
RFXAP	−0.072892459776787
ANLN	0.127880229998519
MYO1E	0.103509906393415
OSGEP	−0.02

## Data Availability

The data supporting the current study are available from the corresponding author upon request.

## Abbreviations

LC: Lung cancer
AGR: Albumin/globulin
SIRI: Neutrophils *∗* Monocytes/lymphocytes
OS: Overall survival
KM: Kaplan–Meier
CEA: Carcinoembryonic antigen
CA199: Carbohydrate antigen 199
BMI: Body mass index
CRP: C-creative protein
SCC: Squamous cell carcinoma antigen
HR: Hazard ratio
TNI: Tumor-nutrition-inflammation index
C-index: Concordance index
ROC: Receiver characteristic operator
NK: Natural killer.

## Additional Points

*What Is Already Known About This Topic?* Answer: It has been reported that low AGR or high SIRI is associated with poorer survival outcome. But these studies focused on a single marker, patients at a certain stage, or a particular cytological classification. *What Does This Article Add?* Answer: This study is the first to combine nutritional, inflammatory, and clinical indicators to establish an integrated biomarker TNI for predicting survival outcomes. It showed that there are some potential molecular mechanisms between neutrophil-albumin-related genes and LC development.

## Supplementary Materials

Supplementary Materials Supplementary figure 1: Kaplan–Meier survival curve of different TNI score groups in validation set (A) and testing set (B). The time-dependent ROC curves of the nomograms compared for 1-year overall survival in patients with advanced lung cancer, respectively. Supplementary figure 2: calibration curves compare predicted and actual survival proportions at 1 year, 2 years, and 3 years, separately. (A) 1 year in training set; (B) 2 years in training set; (C) 3 years in training set; (D) 1 year in validation set; (E) 2 years in validation set; (F) 3 years in validation set; (G) 1 year in testing set; (H) 2 years in testing set; (I) 3 years in testing set. Each point in the plot refers to a group of patients, with the nomogram predicted probability of survival shown on *x* axis and actual survival proportion shown on *y* axis. Distributions of predicted survival probabilities are plotted at the top. Error bars represent 95% confidence intervals. Supplementary figure 3: Kaplan–Meier survival curve of different TNI groups in patients with EGFR mutation (A), patients with non-EGFR-mutation (B), patients who received chemotherapy only as first-line chemotherapy (C), and patients who chosen targeted or immunotherapy regimens as first-line chemotherapy (D). Supplementary table 1: comparison of prognostic performance of three models in training dataset. Supplementary table 2: clinical characteristics of the patients with different TNI groups according to the optimization of cut-off value in total population. Supplementary table 3: univariate and multivariate Cox regression analyses in total population.
