# Supplementary material for: The Prognostic Value and Potential Mechanism of Tumor-Nutrition-Inflammation Index and Genes in Patients with Advanced Lung Cancer
Source: Int J Clin Pract. 2023 May 18;2023:8893670. doi: 10.1155/2023/8893670 (PMC10212685; doi:10.1155/2023/8893670)

Supplementary figure 1: Kaplan-Meier survival curve of different TNI score groups in validation set(A) and testing set(B). The time-dependent ROC curves of the nomograms compared for 1-year overall survival in patients with advanced lung cancer, respectively.

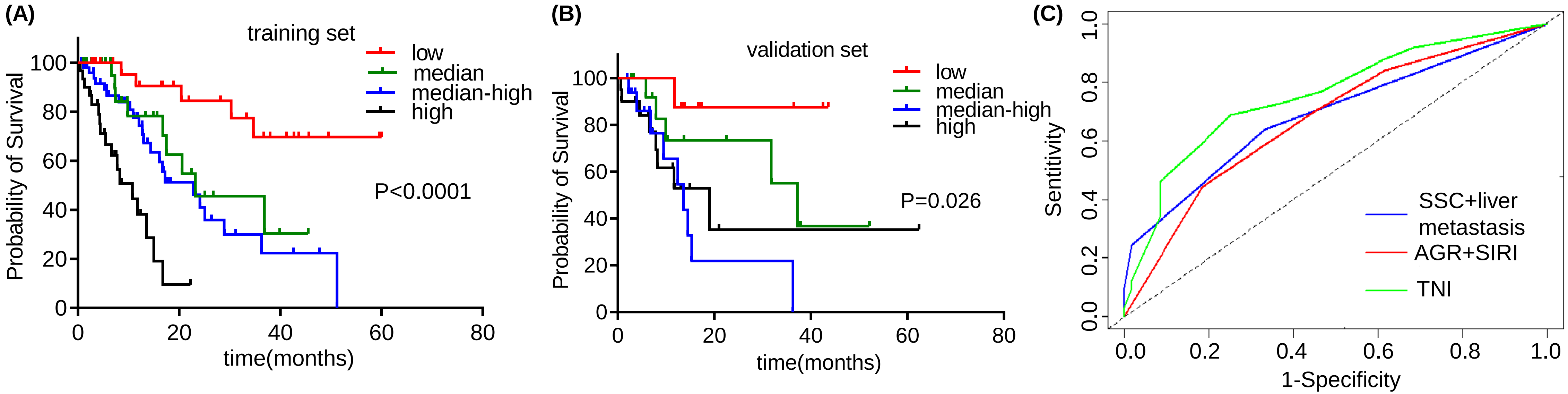

Supplementary figure 2: Calibration curves compare predicted and actual survival proportions at 1 year, 2 years and 3 years, separately. (A)1 year in training set; (B) 2 years in training set; (C)3 years in training set. (D)1 year in validation set; (E) 2 years in validation set; (F)3 years in validation set. (G)1 year in testing set; (H) 2 years in testing set; (I)3 years in testing set. Each point in the plot refers to a group of patients, with the nomogram predicted probability of survival shown on x axis and actual survival proportion shown on y axis. Distributions of predicted survival probabilities are plotted at the top. Error bars represent 95% confidence intervals.

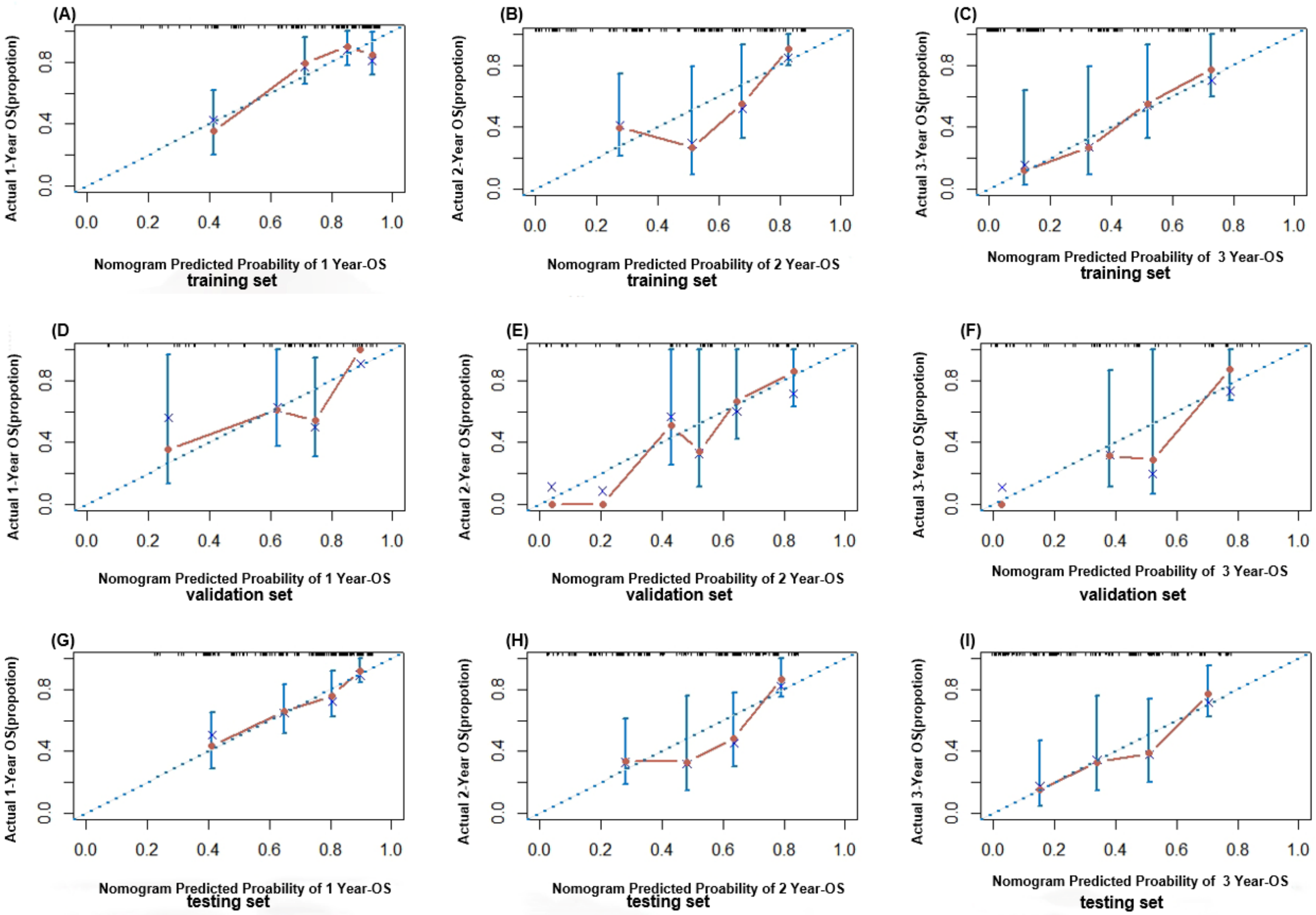

Supplementary figure 3: Kaplan-Meier survival curve of different TNI groups in patients with EGFR-mutation (A), patients with non-EGFR-mutation (B), patients who received chemotherapy only as first-line chemotherapy (C) and patients who chosen targeted or immunotherapy regimens as first-line chemotherapy (D).

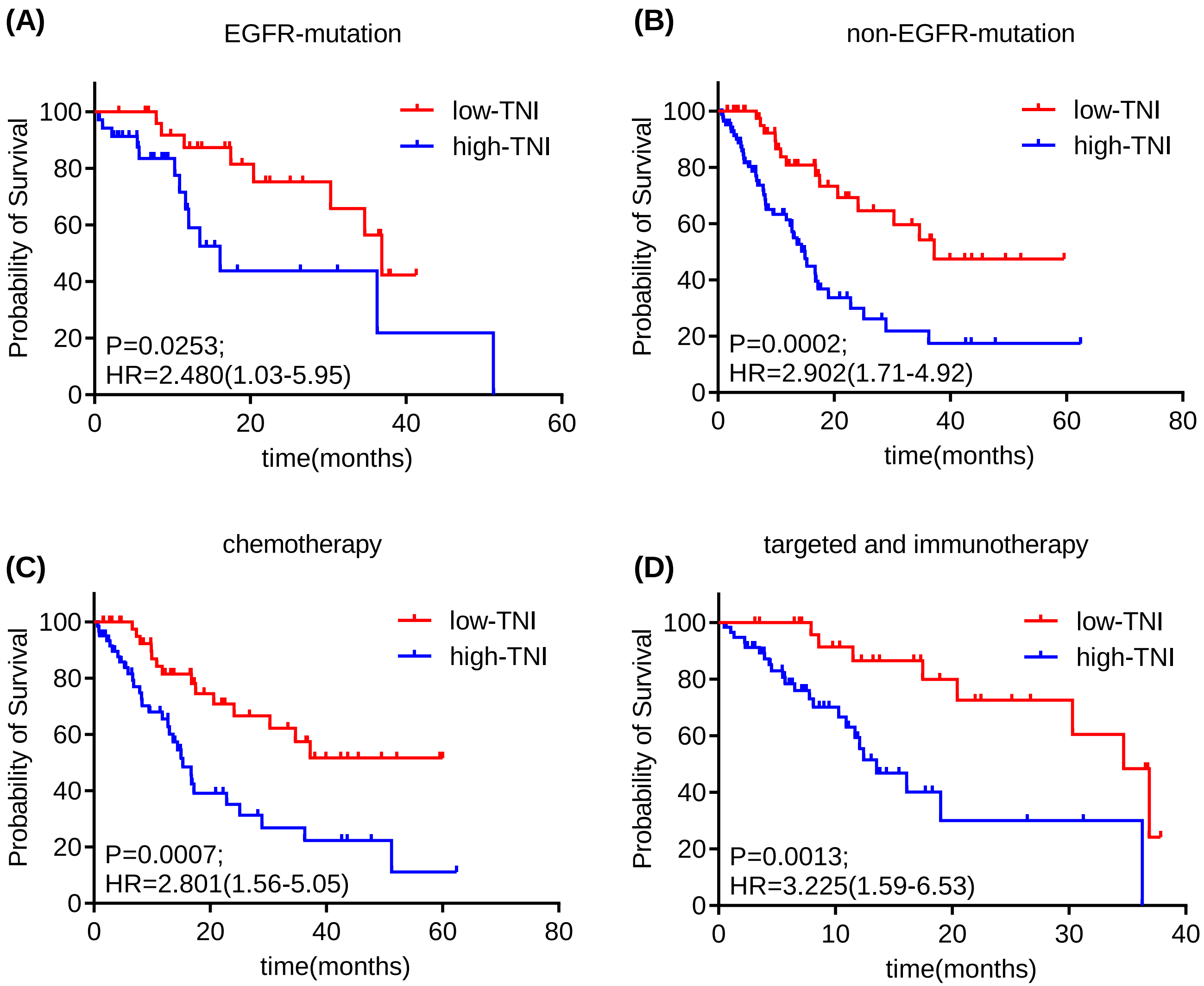

Supplement: Supplementary Materials — Supplementary figure 1: Kaplan–Meier survival curve of different TNI score groups in validation set (A) and testing set (B). The time-dependent ROC curves of the nomograms compared for 1-year overall survival in patients with advanced lung cancer, respectively. Supplementary figure 2: calibration curves compare predicted and actual survival proportions at 1 year, 2 years, and 3 years, separately. (A) 1 year in training set; (B) 2 years in training set; (C) 3 years in training set; (D) 1 year in validation set; (E) 2 years in validation set; (F) 3 years in validation set; (G) 1 year in testing set; (H) 2 years in testing set; (I) 3 years in testing set. Each point in the plot refers to a group of patients, with the nomogram predicted probability of survival shown on x axis and actual survival proportion shown on y axis. Distributions of predicted survival probabilities are plotted at the top. Error bars represent 95% confidence intervals. Supplementary figure 3: Kaplan–Meier survival curve of different TNI groups in patients with EGFR mutation (A), patients with non-EGFR-mutation (B), patients who received chemotherapy only as first-line chemotherapy (C), and patients who chosen targeted or immunotherapy regimens as first-line chemotherapy (D). Supplementary table 1: comparison of prognostic performance of three models in training dataset. Supplementary table 2: clinical characteristics of the patients with different TNI groups according to the optimization of cut-off value in total population. Supplementary table 3: univariate and multivariate Cox regression analyses in total population. [file 8893670.f1.zip › supplementary figures.pdf]
