# Supplementary material for: The Prognostic Value and Potential Mechanism of Tumor-Nutrition-Inflammation Index and Genes in Patients with Advanced Lung Cancer
Source: Int J Clin Pract. 2023 May 18;2023:8893670. doi: 10.1155/2023/8893670 (PMC10212685; doi:10.1155/2023/8893670)
Supplement: Supplementary Materials — Supplementary figure 1: Kaplan–Meier survival curve of different TNI score groups in validation set (A) and testing set (B). The time-dependent ROC curves of the nomograms compared for 1-year overall survival in patients with advanced lung cancer, respectively. Supplementary figure 2: calibration curves compare predicted and actual survival proportions at 1 year, 2 years, and 3 years, separately. (A) 1 year in training set; (B) 2 years in training set; (C) 3 years in training set; (D) 1 year in validation set; (E) 2 years in validation set; (F) 3 years in validation set; (G) 1 year in testing set; (H) 2 years in testing set; (I) 3 years in testing set. Each point in the plot refers to a group of patients, with the nomogram predicted probability of survival shown on x axis and actual survival proportion shown on y axis. Distributions of predicted survival probabilities are plotted at the top. Error bars represent 95% confidence intervals. Supplementary figure 3: Kaplan–Meier survival curve of different TNI groups in patients with EGFR mutation (A), patients with non-EGFR-mutation (B), patients who received chemotherapy only as first-line chemotherapy (C), and patients who chosen targeted or immunotherapy regimens as first-line chemotherapy (D). Supplementary table 1: comparison of prognostic performance of three models in training dataset. Supplementary table 2: clinical characteristics of the patients with different TNI groups according to the optimization of cut-off value in total population. Supplementary table 3: univariate and multivariate Cox regression analyses in total population. [file 8893670.f1.zip › supplementary table 2.docx]

| supplementary table 2 Clinical characteristics of the patients with different TNI according the optimize cut-off in total population | | | | | | |
| --- | --- | --- | --- | --- | --- | --- |
|  |  |  |  |  |  |  |
|  | No. of patients (N=195) | TNI1 (N=33) | TNI2 (N=43) | TNI3 (N=68) | TNI4(N=51) | *P*-value |
| Gender |  |  |  |  |  | 0.001 |
| female | 56(28.7%) | 19(57.6%) | 8(18.6%) | 18(26.5%) | 11(21.6%) |  |
| male | 139(71.3%) | 14(42.4%) | 35(81.4%) | 50(73.5%) | 40(78.4%) |  |
| Age |  |  |  |  |  | 0.533 |
| ≤60 | 53(27.2%) | 9(27.3%) | 15(34.9%) | 15(22.1%) | 14(27.5%) |  |
| ＞60 | 142(72.8%) | 24(72.7%) | 28(65.1%) | 53(77.9%) | 37(72.5%) |  |
| History of LC operation |  |  |  |  |  | 0.001 |
| no | 150(76.9%) | 20(60.6%) | 27(62.8%) | 61(89.7%) | 42(82.4%) |  |
| yes | 45(23.1%) | 13(39.4%) | 16(37.2%) | 7(10.3%) | 9(17.6%) |  |
| Differentiation |  |  |  |  |  | 0.001 |
| poor | 51(26.2%) | 3(9.1%) | 14(32.6%) | 15(22.1%) | 19(37.3%) |  |
| moderate-well | 16(8.2%) | 8(24.2%) | 3(7.0%) | 2(2.9%) | 3(5.9%) |  |
| unknown | 128(65.6%) | 22(66.7%) | 26(60.5%) | 51(75.0%) | 29(56.9%) |  |
| [Pathology](../../%E7%8E%8B%E6%AC%A2/AppData/Local/youdao/dict/Application/7.5.2.0/resultui/dict/?keyword=pathology) |  |  |  |  |  | 0.007 |
| adenocarcinoma | 101(51.8%) | 25(75.8%) | 21(48.8%) | 36(52.9%) | 19(37.3%) |  |
| non-adenocarcinoma | 94(48.2%) | 8(24.2%) | 22(51.2%) | 32(47.1%) | 32(62.7%) |  |
| Mutation |  |  |  |  |  | 0.118 |
| positive | 79(40.5%) | 20(60.6%) | 14(32.6%) | 30(44.1%) | 15(29.4%) |  |
| negative | 58(29.7%) | 7(21.2%) | 13(30.2%) | 19(27.9%) | 19(37.3%) |  |
| unknown | 58(29.7%) | 6(18.2%) | 16(37.2%) | 19(27.9%) | 17(33.3%) |  |
| Bone metastasis |  |  |  |  |  | 0.277 |
| no | 160(82.1%) | 26(78.8%) | 38(88.4%) | 58(85.3%) | 38(74.5%) |  |
| yes | 35(17.9%) | 7(21.2%) | 5(11.6%) | 10(14.7%) | 13(25.5%) |  |
| Brain metastasis |  |  |  |  |  | 0.930 |
| no | 159(81.5%) | 26(78.8%) | 35(81.4%) | 55(80.9%) | 43(84.3%) |  |
| yes | 36(18.5%) | 7(21.2%) | 8(18.6%) | 13(19.1%) | 8(15.7%) |  |
| Adrenal metastasis |  |  |  |  |  | 0.237 |
| no | 182(93.3%) | 30(90.9%) | 43(100.0%) | 63(92.6%) | 46(90.2%) |  |
| yes | 13(6.7%) | 3(9.1%) | 0(0.0%) | 5(7.4%) | 5(9.8%) |  |
| History of smoke |  |  |  |  |  | 0.157 |
| no | 97(49.7%) | 22(66.7%) | 19(44.2%) | 34(50.0%) | 22(43.1%) |  |
| yes | 98(50.3%) | 11(33.3%) | 24(55.8%) | 34(50.0%) | 29(56.9%) |  |
| History of alcohol |  |  |  |  |  | 0.460 |
| no | 146(74.9%) | 25(75.8%) | 36(83.7%) | 49(72.1%) | 36(70.6%) |  |
| yes | 49(25.1%) | 8(24.2%) | 7(16.3%) | 19(27.9%) | 15(29.4%) |  |
| Hypertension |  |  |  |  |  | 0.584 |
| no | 129(66.2%) | 24(72.7%) | 25(58.1%) | 46(67.6%) | 34(66.7%) |  |
| yes | 66(33.8%) | 9(27.3%) | 18(41.9%) | 22(32.4%) | 17(33.3%) |  |
| Diabetes |  |  |  |  |  | 0.461 |
| no | 171(87.7%) | 31(93.9%) | 38(88.4%) | 60(88.2%) | 42(82.4%) |  |
| yes | 24(12.3%) | 2(6.1%) | 5(11.6%) | 8(11.8%) | 9(17.6%) |  |
| BMI |  |  |  |  |  | 0.075 |
| ＜18.5 | 21(10.8%) | 2(6.1%) | 1(2.3%) | 9(13.2%) | 9(17.6%) |  |
| ≥18.5 | 174(89.2%) | 31(93.9%) | 42(97.7%) | 59(86.8%) | 42(82.4%) |  |
| CEA |  |  |  |  |  | 0.470 |
| ≤5 | 86(44.1%) | 18(54.5%) | 20(46.5%) | 26(38.2%) | 22(43.1%) |  |
| ＞5 | 109(55.9%) | 15(45.5%) | 23(53.5%) | 42(61.8%) | 29(56.9%) |  |
| CA199 |  |  |  |  |  | 0.041 |
| ≤43 | 138(70.8%) | 29(87.9%) | 30(69.8%) | 49(72.1%) | 30(58.8%) |  |
| ＞43 | 57(29.2%) | 4(12.1%) | 13(30.2%) | 19(27.9%) | 21(41.2%) |  |
| AFP |  |  |  |  |  | 0.034 |
| ≤7 | 159(81.5%) | 30(90.9%) | 33(76.7%) | 60(88.2%) | 36(70.6%) |  |
| ＞7 | 36(18.5%) | 3(9.1%) | 10(23.3%) | 8(11.8%) | 15(29.4%) |  |
| CRP |  |  |  |  |  | 0.000 |
| ≤5.5 | 84(43.1%) | 30(90.9%) | 22(51.2%) | 16(23.5%) | 16(31.4%) |  |
| ＞5.5 | 111(56.9%) | 3(9.1%) | 21(48.8%) | 52(76.5%) | 35(68.6%) |  |

*LC=lung cancer
