# Supplementary material for: The Prognostic Value and Potential Mechanism of Tumor-Nutrition-Inflammation Index and Genes in Patients with Advanced Lung Cancer
Source: Int J Clin Pract. 2023 May 18;2023:8893670. doi: 10.1155/2023/8893670 (PMC10212685; doi:10.1155/2023/8893670)
Supplement: Supplementary Materials — Supplementary figure 1: Kaplan–Meier survival curve of different TNI score groups in validation set (A) and testing set (B). The time-dependent ROC curves of the nomograms compared for 1-year overall survival in patients with advanced lung cancer, respectively. Supplementary figure 2: calibration curves compare predicted and actual survival proportions at 1 year, 2 years, and 3 years, separately. (A) 1 year in training set; (B) 2 years in training set; (C) 3 years in training set; (D) 1 year in validation set; (E) 2 years in validation set; (F) 3 years in validation set; (G) 1 year in testing set; (H) 2 years in testing set; (I) 3 years in testing set. Each point in the plot refers to a group of patients, with the nomogram predicted probability of survival shown on x axis and actual survival proportion shown on y axis. Distributions of predicted survival probabilities are plotted at the top. Error bars represent 95% confidence intervals. Supplementary figure 3: Kaplan–Meier survival curve of different TNI groups in patients with EGFR mutation (A), patients with non-EGFR-mutation (B), patients who received chemotherapy only as first-line chemotherapy (C), and patients who chosen targeted or immunotherapy regimens as first-line chemotherapy (D). Supplementary table 1: comparison of prognostic performance of three models in training dataset. Supplementary table 2: clinical characteristics of the patients with different TNI groups according to the optimization of cut-off value in total population. Supplementary table 3: univariate and multivariate Cox regression analyses in total population. [file 8893670.f1.zip › supplementary table 3.docx]

| Supplementary table 3 Univariate and multivariate Cox regression analysis in total population | | | | |
| --- | --- | --- | --- | --- |
|  |  |  |  |  |
|  | Univariate |  | Multivariate |  |
|  | HR (95%CI) | P-value | HR (95%CI) | P-value |
| Age | 1.265(0.728-2.197) | 0.404 |  |  |
| Gender | 1.868(1.096-3.185) | 0.022 | 0.934(0.452-1.931) | 0.855 |
| History of LC operation | 0.414(0.224-0.763) | 0.005 | 0.651(0.328-1.291) | 0.219 |
| Pathology | 1.275(0.812-2.003) | 0.291 |  |  |
| Bone metastasis | 1.24(0.693-2.216) | 0.469 |  |  |
| Brain metastasis | 1.067(0.574-1.984) | 0.837 |  |  |
| Adrenal metastasis | 1.307(0.567-3.013) | 0.530 |  |  |
| History of smoke | 1.802(1.136-1.802) | 0.012 | 1.176(0.724-1.912) | 0.513 |
| History of alcohol | 1.268(0.777-2.070) | 0.342 |  |  |
| Hypertension | 0.850(0.517-1.396) | 0.520 |  |  |
| Diabetes | 0.896(0.446-1.798) | 0.757 |  |  |
| BMI | 0.373(0.197-0.704) | 0.002 | 0.506(0.265-0.967) | 0.039 |
| CEA | 1.66(1.037-.657) | 0.035 | 1.373(0.840-2.245) | 0.206 |
| CA199 | 1.279(0.783-2.090) | 0.325 |  |  |
| AFP | 1.068(0.595-1.918) | 0.826 |  |  |
| TNI | Ref | 0.000 | Ref | 0.000 |
| TNI (1/2) | 3.039(1.187-8.082) | 0.021 | 2.575(0.968-6.852) | 0.058 |
| TNI (2/3) | 5.591(2.310-13.535) | 0.000 | 3.390(1.308-8.784) | 0.012 |
| TNI (3/4) | 9.968(3.984-24.943) | 0.000 | 6.891(2.657-17.871) | 0.000 |
| CRP | 3.092(1.883-5.075) | 0.000 | 2.129(1.237-3.664) | 0.006 |

*LC=lung cancer
